# Veterinary Register

**Published:** 1901-07

**Authors:** 


### EDITORIALS.

### VETERINARY REGISTER.

THE JOURNAL proposes to commence in the next issue or two to publish a *Register* of all the veterinarians in the United States and Canada.

Our reasons for entering into this undertaking, which we realize means an enormous amount of work, and which will necessitate a considerable expense for the benefit of the printer, is our desire to bring all the veterinarians in North America into closer relationship, and to show, to those who may not have realized it, what a multiple and powerful body the veterinary profession has become at this beginning of a new century.

The JOURNAL to-day possesses an indexed register of veterinarians far more complete than any list or directory of veterinarians existing in the United States. This list has cost, counting postage and return-postage for undelivered letters and verification of other matter, clerical work, and incidental expenses, not less than $4500 in the last twelve years, and yet it is not complete or satisfactory.

During the last two years in which active work has been done in the cause of an improved army veterinary service, the register of the JOURNAL and lists furnished by the State Secretaries of the A. V. M. A. and by officers of the various State veterinary associations were generously put at the service of the Committee of Army Legislation of the A. V. M. A., and still these lists proved inaccurate.

IN UNION IS STRENGTH. To get union and organization in a body of men we must show to them who their fellow-workers are, and, by showing the herculean proportions of the body of which each veterinarian is a unit, stimulate each to be an important unit of the profession which has just passed its period of infancy in the United States. The veterinary profession has had a hard struggle during the last century to establish itself on a professional basis in this new, developing country.

Such a distinguished, educated, and prominent statesman as the Hon. Mr. Grosvenor, of Ohio, states to the Congress of the United States that "you might as well give rank to the men who curry the colonel's horse as to confer rank upon the veterinary surgeon," while the ruler of England makes the chief veterinarian of the army a Commander of the Bath, and the greatest university of Great Britain confers upon him an LL.D.; and France decorates her prominent veterinarians with the Legion of Honor and makes them members of the Academy.

The Department of Agriculture of the United States Government and many State governments make veterinarians official chiefs and subchiefs of their departmental governments at salaries ranging from $4500 down to $2500, while the army of the United States employs veterinarians as civilian laborers at the wages paid by a brewery or a contractor to their "stable boss."

We realize that a campaign of education and organization has to be carried on, and we propose to lend all the aid we can to it.

In commencing this *Register* of veterinarians we have started with the list in the Commonwealth of Pennsylvania and the State of Ohio, as they are nearest to our office and furnish material for a methodical beginning.

We have issued to each veterinarian in these two States a letter, as follows:

PHILADELPHIA, PA., 1901.

DR. —— ——.

County, Pa. (or Ohio).

DEAR DOCTOR:

We propose in the coming issues of the JOURNAL to publish a complete and accurate *Register* of all the legally qualified veterinarians in Pennsylvania (or Ohio) as they exist now, 1901.

Your name appears on our records as above.

Will you please fill the blanks on the enclosed postal card and return to us at once?

This list is simply to furnish accurate information as to the addresses of qualified veterinarians, and no obligation is incurred by filling the postal.

Yours very truly,
(Signed)
Editor.

With this letter we enclosed a postal card addressed to the JOURNAL, with the following blank form on its reverse:

Write distinctly to avoid any mistake in initials, spelling of name or address.

Name
Street
Town
County
State
College What year
Registered: What County Date

This form will be carried out, and we will send a similar communication to every veterinarian in the United States and Canada.

We desire distinctly to state that the first list published will be based upon the return postal cards.

The JOURNAL bases its authority for publishing the veterinarian's name, address, college (if a graduate), and place and date of registration (where required by law), solely on the information furnished with the signature of each veterinarian.

We do not propose to censor, criticise, or alter in any way the facts as sent us on each postal card.

This will form a basic list of the veterinarians practising in America.

Now, if there be false claims, misstatements, or grounded reasons for corrections we will be glad to have furnished to the profession at large, to the veterinary associations, and to the officials of State Governments interested the data upon which such can be corrected and rectified or upon which more efficient State legislation can be based.

If, as our communications reach each State, any veterinarian finds that any of his colleagues has been overlooked, we will be greatly indebted if the name and address will be furnished us at once.

The JOURNAL'S pages are always open to and invite information, criticism, and all that any colleague may write us over his signature.

